# An Analysis of the Symptoms of Twenty-One Cases of Meningitis in the Adult

**Published:** 1855-04

**Authors:** J. Lewis Smith

**Affiliations:** Physician to the Northwestern Dispensary, New York


*An Analysis of the Symptoms of twenty-one cases of Meningitis in the adult.* By J. LEWIS SMITH, M.D., Physician to the Northwestern Dispensary, New York.—Perhaps there is no inflammatory disease so vaguely written upon, or so little understood, as inflammation of the arachnoid and pia mater. This arises chiefly from the fact that this inflammation usually coexists with that of the dura mater, or cerebral substance, or with disease elsewhere, modifying and obscuring its symptoms, and, perhaps, changing its course.

My purpose, in this paper, is to determine whether the books give a correct account of this disease, and to this end I have collated, as far as possible, records of primary meningitis, and secondary, where the primary disease seemed so mild as not to produce any material modification in the patient's condition.

My investigations have been restricted to adults, from the reflection that in them the symptoms may be different from those in childhood; and in order to avoid those numerous cases of acute hydrocephalus, which is especially a disease of early life.

The whole number in my collection is only twenty-one, as, for various reasons, I have rejected most of the recorded cases of this disease. Of those published in Abercrombie's celebrated treatise, I employ only one; the rest, with the exception of a lady, in whom the disease was combined with inflammation of the ear, being either children, or else showing, after death, merely a vascular condition of the membranes. Vascularity, with or without serous effusion, may indicate, it seems to me, simply a state of congestion; and I find that Dr. Watson, of London, objects to these cases of Abercrombie, on this ground.

The French writers, as Andral, give minute records of meningitis, but most of the cases published by them present such complications, that I have not dared to use them.

Records where the patients recovered, and several such are found in the journals, have also been rejected, in the belief that we cannot yet make a positive diagnosis of meningeal inflammation from the symptoms. Only such cases have been employed as showed after death a lymphy deposit on or under the membranes.

Before proceeding to the analysis, a word should be said of the so-called cerebro-spinal meningitis, an epidemic disease, which has prevailed in various parts of Europe, as Gibraltar and Strasburg, and in our own country. The pathology of this disease is not yet understood,—some considering it a local, others a constitutional affection; and there has been a corresponding discrepancy in the treatment. Whatever may be its nature, it is evidently very distinct from the sporadic inflammation, and cannot properly be considered with it.

In nine of the twenty-one cases, the cause of the disease was not apparent. Perhaps, in some of these, a more minute autopsy would have discovered a morbid process, to which the inflammation was secondary; for the best pathologists agree that secondary meningitis is more common than primary. If, in any of them, such disease were present, it was no doubt mild, to have escaped detection. Five had tubercles in the membranes, in the midst of the inflamed surface, and in four there were tubercles in the lungs, and not elsewhere. Meningeal inflammation has been frequently noticed to accompany phthisis; and as post-mortem examinations often reveal the inflammation, without the presence of tubercles to excite it, the tubercular *diathesis* has been properly called the cause of the meningeal disease. When tubercles are found on the membranes, they, no doubt, in some instances are deposited during the inflammation, just as pneumonia may cause the first tubercular deposition to take place in the central or lower part of the lung, instead of its usual seat, the apex.

In one case, the meningitis seemed to arise from erysipelas of the neck and scalp, in one from intemperance, and in one from reaction after profuse hemorrhage. From the records, it does not appear that the primary and secondary forms differed in any important particular. On the average, the symptoms, both in kind and intensity, seem to have been about the same.

Determining the duration of the disease has been somewhat difficult; but dating from the commencement of well-marked cerebral symptoms, as headaches or delirium, I find, in fifteen cases, the period to vary from one to thirty-three days, with an average of fifteen. In one other case, the time seems pretty accurately fixed at three and a half months, including an interval of improvement.

*Symptoms.*—Headache was one of the most common, generally severe, but sometimes slight. It is recorded in fourteen cases, in all of which it began the first day, and continued till the patients sank into delirium or coma. In no case is its absence recorded.

One only had convulsions. This man was a soldier in the French army at the time of its retreat from Moscow, subsequently to which he was subject to epileptic attacks. An autopsy of all the viscera showed no disease except the meningitis.

How the opinion has become so prevalent, that inflammation of the meninges gives rise to convulsions I do not know, but presume it is because this disease is most common in childhood, and convulsions usually attend this as well as other encephalic diseases in early life. Perhaps English and American physicians have derived their knowledge of diseases of the brain and membranes more from Abercrombie's treatise than any other source; and, as we have said, nearly all the cases in his collection were children. He gives the opinion that "the more common form in which the attack takes place, is by a sudden and long-continued paroxysm of convulsions," alluding to an attack of meningitis. On the contrary, our analysis clearly shows that convulsions are not a symptom of this disease except in childhood, and this correction should be made in our standard works.

A rigid and flexed state of the upper extremities was present in one case, in one trismus, in another paralysis of one side of the face, in another of an arm, and in four of an entire side.

Delirium was noticed in fourteen cases; in three coming on in the commencement of the disease, in the others not till near the close of life. It is not stated whether the remaining seven were delirious, so that if this symptom were present, it was probably of the passive kind.

Vomiting, so common in the acute hydrocephalus of childhood, occurred in only six cases, and in these, with one exception, not till the disease was well advanced.

The pupil in six cases was dilated during the comatose state, and in two others, before the development of coma, it was contracted, the condition during coma not being mentioned. Besides these, four exhibited some unnatural appearance of the eye, as strabismus, occurring, probably, from effusion. In the remaining cases the condition of this organ is not recorded. In one instance where the pupils were dilated, thirty leeches were applied to the neck, and while the bites were still bleeding contraction took place. This goes to show that simple congestion may cause dilation, which may not, therefore, be always so grave a symptom as is usually thought.

Retention of urine was present in six cases, and incontinence in one.

The pulse in seven was under eighty till near the close of life. Of these, three were phthisical, two had headache for two years, and one for life, one had had pain for a considerable time in the lumbar region, the cause not being apparent, and in the other the inflammation appeared to be primary. Three had a pulse varying from 80 to 100, two were phthisical, in the other the inflammation was primary. Three had a pulse over 100, of whom two were consumptives. The thought may occur, whether this discrepancy in the condition of the pulse may not have been due to compression from the effused fluid. A compressed state of the brain, will, in many instances, prevent acceleration of the pulse, though the inflammation is intense. But this explanation does not answer, for the symptoms of compression were generally absent till near the close of life. It is better to consider this diversity due to a difference in the grade of inflammation, as is the case in the inflammation of other serous membranes.

The mode of death in sixteen cases is given, in all by coma, varying from a few hours to two or three days. Generally the effusion of serum and lymph seemed sufficient to cause the coma.

The seat of inflammation in seven cases was the base of the brain, in four the convexity of one hemisphere, in three the upper surface of both, and in two the entire meninges. In the remaining cases the seat of disease was not recorded accurately, though the deposit showed undoubted inflammation.

From this analysis the following conclusions may be drawn:—

1st. That a common cause of meningitis is the tubercular diathesis.

2d. That if in any of these cases the inflammation was primary, and not dependent on a diathesis, it did not differ materially from the secondary form either in gravity or duration.

3d. That meningitis usually commences with headache.

4th. That convulsions are not a symptom of it.

5th. That delirium is present in the majority of cases, occasionally early, but generally not till the disease is far advanced.

6th. Vomiting does not occur till a late stage of the inflammation, and then in only a moderate number of cases.

7th. The pulse differs in different cases, and is, therefore, the less reliable as a means of diagnosis.

8th. Paralysis sometimes occurs at a late stage of the disease, but generally there is no contraction or rigidity of the limbs.

9th. That the mode of death is by coma. It is not our object to speak of the treatment, as all the cases were fatal, and in no instance did the remedies differ materially from those recommended in the books.—*New York Journal of Medicine.*

